# Genome Sequences of Two Bovine Mastitis-Causing *Escherichia coli* Strains

**DOI:** 10.1128/genomeA.00259-15

**Published:** 2015-04-09

**Authors:** Florent Kempf, Valentin Loux, Pierre Germon

**Affiliations:** a INRA, UMR 1282 Infectiologie et Santé Publique, CIRM-Bactéries Pathogènes, Nouzilly, France; b INRA, UMR 1282 Infectiologie et Santé Publique, Immunité et Infections Mammaires, Nouzilly, France; c Université François Rabelais de Tours, UMR 1282 Infectiologie et Santé Publique, Tours, France; d INRA, UR 1077 MIG, Jouy-en-Josas, France

## Abstract

*Escherichia coli* is one of the main pathogenic agents causing inflammatory infections in the bovine udder. Here, we report the draft genome sequences of two strains isolated from different cases of clinical mastitis.

## GENOME ANNOUNCEMENT

Bovine mastitis caused by *Escherichia coli* is a problem of major concern in the dairy industry. This well-known model bacterium has been described as either commensal or pathogenic, depending on the strain and environment considered. Nonetheless, except for some strains involved in recent human epidemic outbreaks, much remains unknown about the genetic background behind the phenotypic diversity of *E. coli* (1). In particular, mastitis strains are generally considered opportunistic pathogens. Several studies have focused on their possible genetic specificities that determine traits that are relevant for pathogenic potential (invasion capacity, virulence, and long-term persistence) (2), but so far, no virulence gene specific for a mastitis-causing *E. coli* isolate has been identified. Interestingly, clinical observations have suggested that mastitis strains might be clustered within a distinct pathotype (mammary pathogenic *E. coli* [MPEC] [3]).

*E. coli* model mastitis strain P4 was recently sequenced (4). In the present study, we considered two strains isolated from cases of clinical mastitis (D6-113.11 and D6-117.07), which may constitute a first corpus for comparative genomics studies. Further studies based on these strains may reveal bovine-associated genetic specificities possibly enabling a wider pathotype definition than that expected under the MPEC hypothesis.

High-quality genomic DNA was extracted using silica-based columns (NucleoSpin tissue; Macherey-Nagel, Hoerdt, France). For each strain, two sequencing runs were performed on an Illumina HiSeq 2000 genome analyzer (GATC, Konstanz, Germany) from a paired-end library and a 3-kb-insert mate-pair DNA library. Paired-end sequencing yielded between 62.7 and 19.6 million reads, totaling 3.196 and 1.031 Gb for D6-117.07 and D6-113.11, respectively. For each strain, the genome coverage was >220×, using the *E. coli* K-12 MG1655 genome size as a reference. Mate-pair library sequencing yielded between 11.3 and 18.9 million reads, totaling 0.057 and 0.963 Gb for D6-117.07 and D6-113.11, respectively (coverage range, 12 to 208×). The initial assembly and read scaffolding were performed using Velvet 1.2.07 (5) and were followed by automatic genome annotation on the AGMIAL platform (6).

The resulting draft genome information is as follows: D6-117.07 has 27 scaffolds and 4,795,662 bp, with a scaffold *N*_50_ of 2,494,862 bp, and D6-113.11 has 48 scaffolds totaling 5,097,170 bp, with a scaffold *N*_50_ of 715,015. Contigs/scaffolds <500 bp were considered potential artifacts and were removed from the study. Automatic annotation yielded 4,557 (D6-117.07) and 4,804 (D6-113.11) nonredundant coding sequences.

The strains are available upon request at the International Center for Microbial Resources—Bacterial Pathogens (CIRM-BP [http://www6.inra.fr/cirm_eng/Pathogenic-Bacteria]) under collection reference numbers and 2IM-260 (D6-117.07) and CIRMBP-549 (D6-113.11).

### Nucleotide sequence accession numbers.

The draft genome sequences have been deposited at the European Nucleotide Archive (ENA) under study accession numbers CCCP00000000 (D6-117.07) and CCCO00000000 (D6-113.11).
